# Two Cases of Immune Thrombocytopenia (ITP) Related to Viral Vector Vaccination ChAdOx1-S (AstraZeneca) and a Good Response after Thrombopoietin Receptor Agonist (TPO-RA) Therapy

**DOI:** 10.3390/hematolrep16040057

**Published:** 2024-09-27

**Authors:** Konstantina Salveridou, Theodoros Tzamalis, Maika Klaiber-Hakimi, Sabine Haase, Stefanie Gröpper, Aristoteles Giagounidis

**Affiliations:** 1 Department of Oncology, Hematology and Palliative Care, Marien Hospital Duesseldorf, 40479 Duesseldorf, Germany; 2 Department of Oncology, Hematology and Palliative Care, Bethesda Hospital Moenchengladbach, 41061 Moenchengladbach, Germany

**Keywords:** ITP, COVID-19, vaccine, TPO-RA

## Abstract

Background: In 2019, a new coronavirus disease emerged in Wuhan, China, known as SARS-CoV-2, severe acute respiratory syndrome coronavirus 2, and caused an ongoing pandemic. Symptomatology of the syndrome is variable, with complications extending to hematopoiesis and hemostasis. Approximately a year after onset of the virus, four vaccination formulas became available to the public, based on a viral vector or mRNA technology. These vaccine formulas have been hampered with hematological complications, like vaccine-induced immune thrombotic thrombocytopenia (VITT) and vaccine-related ITP (immune thrombocytopenic purpura). ITP is a disease with autoimmune pathogenesis characterized by antibody production against platelets and an increased hemorrhagic risk. A decent number of cases have been referred to as possible adverse effects of COVID-19 vaccinations. Case presentation: in this case report, we present two cases of newly diagnosed ITP after vaccination with ChAdOx1-S (AstraZeneca), with a good response to treatment with thrombopoietin-receptor agonists (TPO-RAs). Discussion: we observed an absence of response after corticosteroids and IVIG therapy and a positive therapeutic outcome on TPO-RA. Conclusions: in the ongoing pandemic, there is an urgent need to create therapeutic guidelines for vaccination-related clinical entities and to clarify indications for the vaccination of patients with pre-existing hematological diseases.

## 1. Introduction

In 2019, COVID-19 (coronavirus disease 2019) appeared in Wuhan, China, and was rapidly recognized as a global pandemic and has already infected over 250 million people, causing nearly 5.5 million deaths [1]. Rapid vaccine development ensued based on mRNA technology, and an FDA emergency use authorization was granted to a number of vaccine formulas that have elicited only mild-to-moderate side effects. In a few instances, hematological complications following a COVID-19 vaccine were observed, including a prothrombotic syndrome, characterized as vaccine-induced immune thrombotic thrombocytopenia (VITT) or vaccine-associated (immune) thrombotic thrombocytopenia (VATT) [2,3,4].

Immune thrombocytopenic purpura (ITP) is an acquired thrombocytopenia based on an autoimmune reaction against platelets and megakaryocytes. A distinction is made between a primary form, in which no triggering cause can be identified, and secondary forms, where the immune reaction is due to different pathogenetic mechanisms. The overall incidence of ITP has a spectrum from 2.9 to 3.3/100.000 persons, with a female preponderance and a higher incidence among children and people > 60 years of age [5,6]. ITPs linked to vaccination have an incidence of around 1% [7].

Cases of worsening ITP, possibly secondary to the mRNA-based COVID-19 vaccine, have incited scientific interest. For example, according to recent studies, some patients with chronic ITP might have a transient exacerbation of their thrombocytopenia within one week of their COVID-19 vaccination [8].

Vaccines, in general, have the potential to create autoimmune reactions, including type 1 diabetes mellitus, multiple sclerosis, Guillain-Barré syndrome, and acute disseminated encephalomyelitis [9].

Vaccine-induced thrombocytopenia has already been observed, e.g., in children 13–24 months old, who were reported with newly diagnosed ITP during the first six weeks following measles–mumps–rubella (MMR) vaccination [10]. Furthermore, ITP is a known side effect after vaccination against influenza, varicella, herpes zoster, haemophilus influenza, and hepatitis B virus (HBV) [9,11,12,13,14].

Other studies have highlighted the effects of COVID-19 infection on primary hemostasis (i.e., platelets, von Willebrand factor, and endothelium), as well as secondary hemostasis and fibrinolysis [15,16,17,18]. Several mechanisms involving endothelial dysfunction and pathogenic autoimmune responses, subsequent to a generalized inflammatory response during a COVID infection, can induce hematopoietic disturbances, thrombotic events, or even the production of autoantibodies [19,20]. In the case of COVID-19 vaccines, similar mechanisms based mainly on molecular mimicry have been incriminated.

We report two cases of immune thrombocytopenia occurring 1–2 weeks after receiving a first vaccination with the AstraZeneca COVID-19 vaccine, with both showing a good response to TPO-RA therapy.

## 2. Case Report 1

A 54-year-old healthy male received his first COVID-19 vaccination with the AstraZeneca vaccine in March 2021. He had no pre-existing medical history and no history of adverse reactions after previous vaccinations. A routine check about one year before the vaccination revealed a platelet count at the lower end of normal limits (157 × 10^3^/µL). He presented to his GP on 10 May 2021, reporting a significant tendency to bruise during the last month, two weeks after receiving the first vaccination against COVID-19.

Laboratory tests revealed an unexplained thrombocytopenia of 12,000/µL, and he was referred to a local hospital on May 2021 for further investigation. A physical examination showed petechiae and small hematomas. Vital signs and the remainder of his exam were non-contributory. He denied a recent history of infection. An abdomen ultrasound scan showed an image of hepatic steatosis without signs of splenomegaly. An immunological assay revealed no anti-platelet antibodies in serum or plasma. Autoantibodies against platelet factor 4 (PF4) were also not detected. He tested negative for Helicobacter pylori colonization. Laboratory tests revealed a normal white-cell count, hemoglobin, and severe thrombocytopenia with a platelet count of 2000/µL. The following labs were within normal limits: prothrombin time, activated partial thromboplastin time, fibrinogen, creatine, electrolytes, aspartate aminotransferase, alanine aminotransferase, bilirubin, LDH, alkaline phosphatase, albumin, immune fixation, total protein, ANA, ANCA antibodies, and haptoglobin. Additionally, he tested negative for HIV, hepatitis B, hepatitis C antibody, and Epstein–Barr virus serology. Bone marrow cytology showed findings consistent with immune thrombocytopenia. A nasopharyngeal swab also returned as negative for SARS-CoV-2 antigen. In the absence of clinical findings and due to normal D-dimer levels, no diagnostic imaging was performed in order to exclude a thromboembolic event (Table 1).

**Figure 1:**
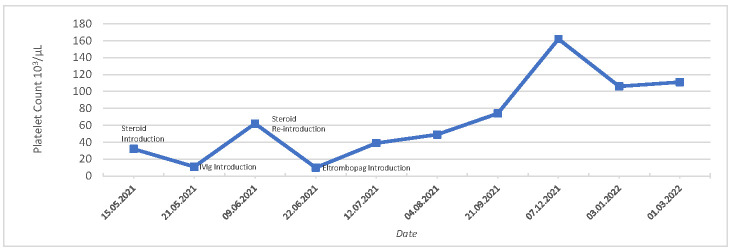
Platelet counts over time and response to treatment. Case Report 1.

Given a probable diagnosis of immune thrombocytopenia, the patient received dexamethasone pulse therapy 40 mg/day for 4 days as a first-line therapy in May, which led to a rise in platelets to 63,000/µL within two weeks (Figure 1). However, the patient complained of strong singultus after taking dexamethasone, whereupon the treatment was terminated. In a follow-up appointment, a sharp drop in the platelet count to 11,000/µL was noted and immunoglobulin (IVIG) therapy was initiated. He received a cumulative dose of IVIG 80 g over two days, and the platelet count stabilized at approximately 25,000/µL. Additional dexamethasone pulse therapy led to an increase in platelet count followed by a rapid drop to 8000/µL. Due to an inadequate response, we switched to a TPO-receptor agonist therapy with eltrombopag at a dose of 25 mg 1× daily. The patient received his second COVID-19 vaccination with BioNTech a month later. No significant adverse reactions or fluctuations in platelet count were noted. The dose of eltrombopag was increased to 50 mg/day, and the platelet count reached 39,000/µL after the second vaccination. A further increase to 75 mg eltrombopag was performed, and two months later, the platelet count had normalized to 160,000/µL (Figure 1). Eltrombopag was slowly tapered and discontinued in January 2022 (Table 1). The patient has remained off therapy since, displaying normal platelet counts.

## 3. Case Report 2

A 60-year-old male with a history of autoimmune diseases presented with worsening petechiae and ecchymoses on his lower extremities two weeks after receiving his first dose of the AstraZeneca SARS-CoV-2 vaccine. As mentioned above, he had a history of autoimmune disease with antecedent atopic dermatitis, pulmonary sarcoidosis, multiple food allergies, and Hashimoto’s thyroiditis. Laboratory tests revealed pronounced thrombocytopenia (1000 platelets/µL) with no further significant laboratory abnormalities, LDH values in normal range, and normal D-dimer levels. Inflammatory markers were not increased either (Table 1). Shortly after his first vaccination, he developed a fever and common flu symptoms. Examination of a peripheral blood smear revealed a decreased number of platelets with no signs of platelet aggregation or dysplasia in neutrophils and erythrocytes, which are findings compatible with immune thrombocytopenia. A bone marrow examination revealed a rather abundant number of megakaryocytes, which, in combination with peripheral thrombocytopenia, possibly resembles clinical presentation of ITP. Our patient underwent serological testing for rheumatological diseases, which returned no indicting findings.

**Figure 2:**
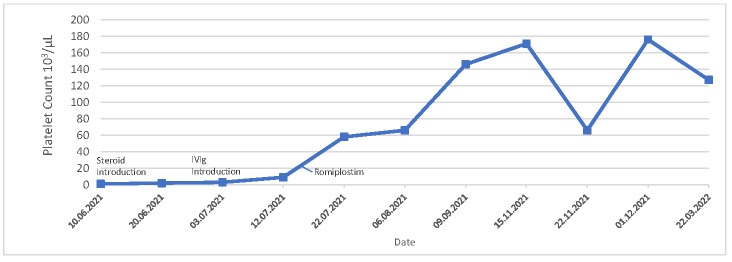
Platelet counts over time and response to treatment. Case Report 2.

He tested negative for HIV, hepatitis B virus, hepatitis C virus, Epstein–Barr virus, cytomegalovirus, and parvovirus B19. Investigations for a broad range of autoantibodies remained negative, including antibodies against PF4. A brain CT scan revealed no indications of an infarction or thrombosis. He received 40 mg of dexamethasone for 4 days, followed by an intravenous immune globulin regime (0.4 g/kg/day) for 5 days with an inadequate platelet response, ranging from 1000 to 3000/µL (Figure 2). He was then initiated on the thrombopoietin receptor agonist Romiplostim (1 µg/kg/week). The patient responded within a week of therapy initiation, and this was continued on adjusted doses until a stable platelet count ≥ 50,000/µL was achieved for at least 4 weeks without dose adjustment (Figure 2). He continued treatment up to a dose of 10 µg/kg/week. As of the time of writing, the patient remains on Romiplostim therapy (10 µg/kg/week), maintaining his platelet response (Table 1).

## 4. Discussion

We present two additional cases of purportedly secondary ITP following a ChAdOx1 nCOVID-19 vaccine. An important part of our workup was to rule out a differential diagnosis of VITT (vaccine-induced immune thrombotic thrombocytopenia), which is a syndrome related to vaccination against SARS-CoV-2 [8,21,22]. Apart from a low platelet count, VITT presents with clinical signs of venous or arterial thrombosis, including atypical sites, such as sinus venous thrombosis. The main laboratory diagnostic key is the detection of anti-PF4 antibodies, which were negative in our cases. Furthermore, VITT is typically associated with a slightly prolonged PT or aPTT, and a low fibrinogen value, as well as increased D-dimers. [21,22,23]. All of the above-mentioned markers remained within the normal range for our patients. 

Both patients were refractory to, or had insufficient response to, conventional ITP therapy. Both responded to TPO-RA therapy. A previous medical history of thrombocytopenia or other hematological disease was not noted in either case. The short interval of appearance of thrombocytopenia after vaccination and the lack of alternative explanations makes vaccine-induced ITP highly likely. Although one could argue that drug-induced thrombocytopenia should respond to immunosuppressive therapy, both patients had only minor responses to dexamethasone pulse therapy or treatment with immunoglobulins. But we have seen cases with a good response to corticosteroids. Similar cases have also been reported [22,23,24].

It would be interesting to investigate a possible link between the pathogenesis of ITP, in our case a possible vaccine-induced pathomechanism, with the efficacy of the different therapeutic regimes, so that a better assessment can be made regarding in which cases the therapy is effective.

Vaccine-induced ITP has previously been reported following vaccination [23]. A drug induced mechanism has been already investigated [23,25]. Associations with other vaccines, such as hepatitis A and B, diphtheria–tetanus–acellular pertussis (DTaP), and varicella and Shingrix recombinant Zoster vaccine, are recognized [11,13,14]. Vaccines may trigger several autoimmune mechanisms in the wake of induction of protective immunity. These include molecular mimicry, polyclonal activation, bystander activation, and the presence of super-antigens. As an example, molecular mimicry is based on the similarity between a vaccine’s epitope with a self-antigen, which results in an autoimmune reaction with IgM antibodies aimed at platelet surface antigens. In the case of polyclonal activation, a massive B-cell activation leads to increased antibody and immunoglobulin production, mainly IgG, which may attack healthy tissues [26,27,28,29,30].

Other pathogenetic mechanisms important for cell-mediated immunity and phagocyte-dependent inflammation, such as those mediated by CD4 helper Th1 cells, have been implicated. Studies have shown how T-cell defects resulting in an imbalanced self-tolerance may play a role, especially in chronic ITP patients. A T-cell mediated mechanism incorporating the production of pro-inflammatory cytokines, such as IFN-γ and TNF, and chemokines, such as CXCL10, may play a role in vaccine-induced cases. Specifically, B-lymphocyte stimulator (BLyS), which is a soluble ligand of the TNF cytokine family, has been related to ITP [31,32]. The pathogenesis of a vaccine-induced autoimmune disease can possibly be due to the cytokine milieu. Cytokines are indicators of inflammation, which are not systematically determined in clinical practice. A small fraction of cytokines are actually investigated in clinical practice for detecting various inflammation syndromes. Due to their short in vivo half-life, these are not indicative values of the current immune balance, or objective diagnostic markers, even for syndromes such as CRS (cytokine release syndrome). A cytokine release has most likely taken place in patients due to previous vaccination. A possible connection may exist but is not conclusive, so the cytokine milieu may not have clinical relevance at the point of diagnosis.

Furthermore, adjuvants used for enhancement of the immunological response like aluminum salts, DNA, and lipids may also play a role [30,33,34]. Schoenfeld et al. described a new autoimmune syndrome (ASIA) induced by adjuvants [29]. 

Cases of ITP secondary to a COVID-19 infection have already been published. Bhattacharjee et al. presented a systemic review of 45 patients with ITP associated with a COVID-19 infection [35]. RNA-related treatments have been linked with hematological complications. Severe thrombocytopenia has been observed with a small number of antisense oligonucleotides [36,37]. Furthermore, there have also been reports of ITP following mRNA-based COVID-19 vaccines. The exact mechanism of ITP induction in these cases remains to be elucidated. One hypothesis would be that individuals with an immunological predisposition or a compensated hereditary or idiopathic ITP might be especially prone to developing the full picture of the disease [38,39]. 

According to a national prospective cohort in Scotland, an estimated incidence of 1.13 (0.62–1.63) ITP cases per 100,000 doses was reported, with the onset fluctuating from one week to 21–27 days post-vaccination [21].

Recent studies indicate how mRNA can lead to a massive activation of immune cells, resulting in cytokine and chemokine secretion through the upregulation of toll-like receptors [40]. Lipid nanoparticles, which are responsible for mRNA delivery, have also been accused. Specifically, the mRNA-LNP used in preclinical vaccine studies can be highly inflammatory. The nucleoside-modified mRNA-LNP platform’s lipid nanoparticle component used by Pfizer/BioNTech and Moderna in their SARS-CoV-2 vaccines has been accused of inducing a highly inflammatory response in mice, inducing IL-1β and IL-6 production [41].

Other cases of thrombocytopenia following COVID vaccination may be manifestations of another rare adverse reaction like VITT (vaccine-induced immune thrombotic thrombocytopenia). It is characterized as the detection of anti-heparin/PF4 antibodies [23].

Secondary ITP linked to COVID vaccines appears to have a more favorable prognosis. A study conducted with fifty-two consecutive chronic ITP patients after COVID-19 vaccination showed that 12% had an exacerbation within 2–5 days post-vaccination with symptoms and with a good response after rescue therapy with corticosteroids +/−IVIG [8]. Thrombopoietin receptor agonists (e.g., Romiplostim and eltrombopag) are indicated as a second-line treatment and have demonstrated improved platelet counts [8]. Our cases are notable for the absence of a response after corticosteroids and IVIG therapy and for their sustained response on TPO-RA.

## Figures and Tables

**Table 1:** Overview table.

		Case Report 1	Case Report 2
**Vaccine**		ChAdOx1 nCOVID-19	ChAdOx1 nCOVID-19
Symptoms	Petechiae	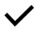	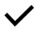
	Hematomas	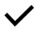	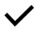
Laboratory test	Anti-platelet antibodies	WNR	WNR
	Anti-PF4 antibodies	WNR	WNR
	Prothrombin time (PT)	WNR	WNR
	Partial thromboplastin time (PTT)	WNR	WNR
	D-dimers	WNR	WNR
	Fibrinogen	WNR	WNR
	Platelet count	1000/µL	12,000/µL
	White blood cell count	WNR	WNR
	Hemoglobin	WNR	WNR
	Haptoglobin	WNR	WNR
	Virus serology *	negative	negative
	Lactate dehydrogenase (LDH)	WNR	WNR
Therapy	Dexamethasone pulse therapy	40 mg/day for 4 days (repeated twice)	40 mg/day for 4 days
	IVIG therapy	80 g over 2 days	0.4 g/kg/day over 5 days
	TPO-receptor agonist	Eltrombobag 25–75 mg/day	Romiplostin 1–10 µg/kg/week
	Duration of therapy	6 months	ongoing to date

WNR: within normal range; * HIV, hepatitis B virus, hepatitis C virus, Epstein–Barr virus, cytomegalovirus, and parvovirus B19.

## Data Availability

The data that support the findings of this study are available from the corresponding author upon reasonable request.
